# Amyloid β-Induced Inflammarafts in Alzheimer’s Disease

**DOI:** 10.3390/ijms26104592

**Published:** 2025-05-10

**Authors:** Shihui Ding, Soo-Ho Choi, Yury I. Miller

**Affiliations:** Department of Medicine, University of California, San Diego, CA 92093, USA; s9ding@health.ucsd.edu (S.D.); soc002@health.ucsd.edu (S.-H.C.)

**Keywords:** amyloid beta, inflammarafts, lipid rafts, inflammation, Alzheimer’s disease, microglia, reactive oxygen species, apolipoprotein A-I binding protein

## Abstract

The formation of amyloid beta (Aβ) plaques is a central process in the development of Alzheimer’s disease (AD). Although its causative role or the effectiveness of therapeutic targeting is still debated, the key involvement of Aβ in the pathogenesis of neuroinflammation and neurodegeneration in AD is broadly accepted. In this review, we emphasize the role of lipid rafts, both in APP cleavage producing Aβ in neurons and in mediating Aβ inflammatory signaling in microglia. We introduce the term inflammarafts to characterize the Aβ-driven formation of enlarged, cholesterol-rich lipid rafts in activated microglia, which support protein–protein and lipid–protein interactions of inflammatory receptors. Examples reviewed include toll-like receptors (TLR2, TLR4), scavenger receptors (CD36, RAGE), and TREM2. The downstream pathways lead to the production of cytokines and reactive oxygen species, intensifying neuroinflammation and resulting in neuronal injury and cognitive decline. We further summarize emerging therapeutic strategies and emphasize the utility of apolipoprotein A-I binding protein (AIBP) in selective targeting of inflammarafts and attenuation of microglia-driven inflammation. Unlike the targeting of a single inflammatory receptor or a secretase, selective disruption of inflammarafts and preservation of physiological lipid rafts offer a novel approach to targeting multiple components and processes that contribute to neuroinflammation in AD.

## 1. Introduction

Life expectancy has increased in modern societies; however, this has also led to a rapid rise in the number of individuals affected by dementia. Among various causes of dementia, Alzheimer’s disease (AD) is the most prevalent, currently affecting close to 55 million individuals worldwide—a number projected to reach 132 million by 2050 [1]. In the United States, AD remains the fifth-leading cause of death among Americans aged 65 and older, with an estimated 6.9 million individuals in this age group living with the disease [2].

The importance of the amyloid cascade in the pathogenesis of AD is strongly supported by substantial genetic and experimental studies [3,4,5]. In this cascade, amyloid precursor protein (APP) is cleaved to produce a collection of amyloid beta (Aβ) peptides, which aggregate, forming plaques and disrupting normal brain function. Aβ accumulation serves as the primary trigger for a cascade of molecular events leading to neuroinflammation, the activation of neuronal death mechanisms, and ultimately, dementia [5]. The amyloid–neuroinflammation hypothesis originated from histopathological studies showing the presence of pro-inflammatory activated microglia at sites of amyloid deposition in human AD brains [6], with evidence suggesting that anti-inflammatory therapy alleviates symptoms. Thus, epidemiological studies indicate that the long-term use of non-steroidal anti-inflammatory drugs (NSAIDs) can reduce the risk of developing AD, particularly in individuals treated during middle age [7,8]. While Aβ has been the prime target for AD therapy, the repeated underwhelming results of Aβ-targeted clinical trials have raised significant doubt on whether current drug development strategies are on the right path [9].

**Figure 1:**
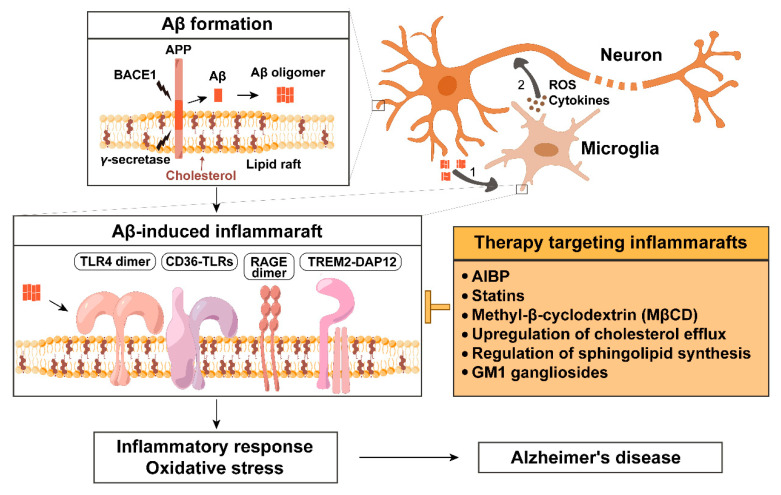
Amyloid β-induced inflammaraft formation and its therapeutic targeting in Alzheimer’s disease. In neurons, amyloid precursor protein (APP) is processed by β-secretase BACE1 and γ-secretase within lipid rafts to generate amyloid beta (Aβ), which can form oligomers. (1) Aβ oligomers spread, bind to microglial surface receptors and membrane lipids (such as cholesterol and gangliosides), and promote the formation of enlarged, cholesterol-rich microdomains termed inflammarafts. Inflammarafts are the membrane platforms for TLR4 dimerization, the assembly of CD36-TLR heterodimers (CD36-TLR4/6 and CD36-TLR2/1), RAGE dimerization, and the formation of TREM2-DAP12 signaling complexes. (2) Activated microglia subsequently release pro-inflammatory cytokines and reactive oxygen species (ROS), which propagate inflammatory signaling and oxidative stress in surrounding neurons, contributing to neurodegeneration.

In this article, we consider an alternative to direct targeting Aβ production or clearance from the brain. A common feature of Aβ production and the pathological effects Aβ mediates is the requirement of key process components to be localized to lipid rafts (Figure 1). The lipid raft localization of APP, β-secretase and γ-secretase, the substrate and enzymes producing Aβ, is well documented in the amyloidogenic pathway [10]. Less appreciated are the findings that the Aβ engagement of inflammatory receptors and Aβ binding to membrane lipid components mediate the enlargement of lipid rafts, creating a stable platform for protein complex assembly, which is required for initiating inflammatory signaling. We call these enlarged, pathological lipid rafts in inflammatory cells “inflammarafts” [11]. The formation of inflammarafts has been associated with conditions such as atherosclerosis, neuropathic pain and glaucomatous neuroinflammation [12,13,14,15]. Increasing evidence implicates lipid rafts in Aβ production and toxicity [10,16,17]. When Aβ interacts with microglia, it recruits lipid raft components and activates toll-like receptor 4 (TLR4), triggering inflammatory response and oxidative stress [18]. Given the limitations of Aβ-targeted therapies or long-term NSAID use, inflammarafts represent a promising alternative therapeutic target in Alzheimer’s disease.

Therapeutic strategies targeting inflammarafts include apolipoprotein A-I binding protein (AIBP), cholesterol-modulating agents (such as statins, β-cyclodextrins, activation of liver X receptors, and upregulation of the cholesterol transporters ABCA1 and ABCG1), the regulation of sphingolipid synthesis, and the administration of GM1 gangliosides. These interventions aim to disrupt inflammaraft formation and mitigate neuroinflammation.

## 2. Aβ Biogenesis Depends on Lipid Rafts

Aβ is a proteolytic fragment derived from the amyloid precursor protein (APP). APP can be processed via two distinct pathways: the non-amyloidogenic and the amyloidogenic pathway. In the non-amyloidogenic pathway, APP is cleaved by α-secretase within the Aβ domain, which prevents Aβ generation. This cleavage yields a soluble ectodomain (sAPPα) and a membrane-bound C-terminal fragment (α-CTF). Subsequent cleavage of α-CTF by γ-secretase produces a truncated, non-toxic peptide known as p3, and the APP intracellular domain (AICD). In contrast, the amyloidogenic pathway begins with β-secretase (BACE1) cleavage of APP, generating sAPPβ and the membrane-bound β-CTF. When γ-secretase acts on β-CTF, it produces Aβ peptides—primarily Aβ40 and the more aggregation-prone Aβ42, which is more hydrophobic and is the predominant species found in senile plaques in AD [3,19,20,21].

Lipid rafts are a metaphor for liquid-ordered membrane microdomains enriched in cholesterol and sphingolipids. Lipid rafts exhibit tightly packed lipids due to the cholesterol–sphingolipid interaction and the saturated acyl chains of sphingolipids, resulting in increased membrane rigidity and restricted lateral diffusion compared to the surrounding non-raft, liquid-disordered regions. Functionally, lipid rafts compartmentalize various cellular processes by anchoring proteins and linking the membrane to the cytoskeleton and extracellular matrix. This structural role facilitates communication between the intracellular and extracellular environments, particularly in response to extracellular signaling [17,22]. Lipid rafts can be stabilized into larger platforms through protein–protein and protein–lipid interactions [23].

Lipid rafts regulate APP processing by enhancing its interaction with BACE1 and promoting Aβ production. Both β- and γ-secretases exhibit higher activity in lipid rafts, where cholesterol stabilizes these enzymes and prevents their degradation. In contrast, α-secretase cleavage typically occurs outside of lipid rafts, in more fluid, cholesterol-poor regions of the membrane [24].

Under physiological conditions, α-secretase cleavage predominates over β-secretase cleavage and occurs in non-lipid raft areas. While secretases are relatively stably localized, APP exhibits dynamic distribution across the plasma membrane, endosomes, endoplasmic reticulum, Golgi apparatus and sub-compartments such as mitochondria-associated ER membrane (MAM), where Aβ production also occurs [24,25]. Normally, only a small, but physiologically relevant, portion of APP is located in lipid rafts [26]. However, the APP localization to lipid rafts is particularly important for Aβ generation (Figure 1).

Enrichment of lipid raft components, such as cholesterol and gangliosides, has been shown to promote Aβ production [16]. In particular, high cholesterol levels shift APP localization toward lipid rafts, favoring amyloidogenic cleavage and suppressing α-secretase activity [27]. Similarly, palmitoylation of APP promotes its localization to lipid rafts, thereby enhancing amyloidogenic processing. Altering APP palmitoylation levels directly affects Aβ production, with increased palmitoylation enhancing Aβ generation, while decreased palmitoylation reduces it [28].

## 3. Aβ Associated Inflammatory Pathways

As early as the 1990s, postmortem studies identified pronounced microglial activation in the brains of AD patients, especially surrounding Aβ-containing plaques [29,30]. Ultrastructural analyses revealed individual microglia extending their filopodia into the core of plaques [31], pointing to a direct interaction between microglia and Aβ and suggesting that Aβ accumulation may serve as a major driving force for microglial activation. Furthermore, in vivo positron emission tomography studies have confirmed this association, showing increased activated microglial load in AD patients, and this increase was directly correlated with cognitive decline [32].

In the healthy brain, microglia remain in a surveillance or homeostatic state, constantly monitoring the environment to maintain homeostasis [33]. Under pathological conditions, however, they become activated in response to various stimuli. The activated microglia release pro-inflammatory cytokines—such as tumor necrosis factor alpha (TNF-α), interleukin 6 (IL-6), and IL-1β—as well as cytotoxic molecules like nitric oxide (NO) and reactive oxygen species (ROS), contributing to the elimination of pathogens but also causing damage to healthy neurons. In contrast, homeostatic microglia secrete anti-inflammatory cytokines such as IL-10 and TGF-β, promoting the resolution of inflammation and supporting tissue repair [34].

In the AD brain, chronic microglial activation is consistently observed in proximity to Aβ plaques [35], and the release of pro-inflammatory factors from these activated cells is believed to exacerbate disease progression [36]. However, microglial responses to Aβ are highly complex and appear to play both protective and detrimental roles. On one hand, microglia can phagocytose and degrade Aβ. On the other hand, prolonged exposure to Aβ triggers the release of a broad range of neurotoxic molecules, such as pro-inflammatory cytokines, chemokines, complement proteins, and reactive oxygen and nitrogen species, which contribute to synaptic dysfunction, myelin damage, neuronal cell death and cognitive impairment [34].

This dual nature of microglial activation underscores the complexity of their role in AD. While early activation may serve a protective function, sustained or dysregulated activation likely shifts the balance toward neurotoxicity. Emerging evidence suggests that microglial dysfunction—characterized by impaired Aβ clearance and enhanced inflammatory signaling—amplifies neurodegeneration in AD. If this hypothesis holds true, restoring microglial homeostasis or modulating their activation state may offer a promising therapeutic approach for slowing or halting AD progression [21].

## 4. Inflammarafts in Activated Microglia and Macrophages

Recent advances in single-cell sequencing have identified several populations of activated and homeostatic microglia [37,38]. In addition to varying transcriptional and epigenetic profiles, microglia also differ in the architecture of lipid rafts. The term inflammarafts refers to enlarged lipid rafts that serve as scaffolds for organizing inflammatory signaling. Inflammarafts are characterized by several key features: an increased number and size of lipid rafts in inflammatory cells, higher cholesterol content per raft, elevated levels of inflammatory receptors, adaptor molecules, ion channels, and enzymes, and crucially, the formation of multimeric protein complexes within the membrane. One of the defining features of inflammarafts is TLR4 dimerization, a key step in initiating inflammatory signaling cascades [11]. The dimerization of TIR domains in TLR4 molecules recruits Mal/MyD88 and TRAM/TRIF adaptor molecules, which, through several intermediates, activate nuclear factor κB (NF-κB), mitogen-activated protein kinases JNK, p38, ERK1/2, and interferon regulatory factor 3 (IRF3), resulting in the expression of inflammatory cytokines and interferon-inducible genes. Woller et al. (2018) demonstrated that inflammatory stimulation leads to increased cellular binding of cholera toxin B to GM1 gangliosides (a lipid raft marker), elevated cholesterol content in isolated rafts, significantly increased TLR4 occupancy in lipid rafts and, importantly, TLR4 dimerization within these domains—all hallmark indicators of inflammaraft formation [39].

In pathological conditions such as chemotherapy-induced peripheral neuropathy (CIPN), TLR4 is localized to enlarged, cholesterol-rich inflammarafts in spinal microglia, driving neuroinflammatory responses [40]. Similarly, macrophages harboring inflammarafts maintain a chronic pro-inflammatory state and are primed for exaggerated responses to additional stimuli. In mouse models of atherosclerosis, inflammarafts are predominantly observed in non-foamy macrophages—the primary inflammatory population—whereas lipid-laden foam cells exhibit less inflammarafts and suppressed inflammatory signaling, highlighting the key role of inflammarafts in maintaining immune activation [13,14]. The inflammaraft-related mechanisms learned from these studies will be useful to test the relevance of the inflammaraft hypothesis to AD pathogenesis and identify novel therapeutic targets.

## 5. Regulation of Inflammaraft and Its Components by Aβ

Lipid rafts serve as signaling hubs where Aβ interacts with multiple receptors, including TLR4 and TLR2, scavenger receptors CD36 and RAGE, and TREM2. These interactions activate downstream inflammatory pathways and promote Aβ internalization or clearance. Many of these receptors contribute to chronic inflammation and further increases in Aβ production, creating a pathological feedback loop [41]. In addition, Aβ affects lipid raft organization by altering the composition and distribution of membrane lipids and receptors. By modulating inflammaraft components and receptor signaling, Aβ not only triggers neuroinflammation but also amplifies its own toxicity and accumulation, contributing to AD progression. In this section, we will review effects of Aβ on major protein and lipid components of inflammarafts.

### 5.1. TLR2 and TLR4

Toll-like receptors are increasingly recognized as key mediators in AD pathogenesis. Elevated levels of TLRs mRNA have been detected in the brain of AD patients, suggesting an active role of TLRs in neuroinflammation [42]. Human microglia express TLRs 1–9, and Aβ is recognized as a danger-associated molecular pattern (DAMP) that activates TLRs—especially TLR2 and TLR4—leading to downstream NF-κB and MAPK signaling, cytokine release, and phagocytosis [43].

Elevated TLR2 expression has been reported in both AD patients and animal models [44]. Aβ oligomers and fibrils activate TLR2-dependent responses in microglia; in TLR2-deficient mice, Aβ42 fails to upregulate immune markers such as iNOS and CD11b [45]. Blocking TLR2 function with antibodies or genetic deletion attenuates Aβ-induced cytokine production, shifts microglia toward a homeostatic phenotype, enhances brain-derived neurotrophic factor (BDNF) expression, and improves neuronal outcomes in APP/PS1 mice (transgenic mice expressing a chimeric mouse/human APP and a mutant human presenilin 1) [46,47]. Moreover, TLR2 deletion facilitates Aβ clearance, likely through increased microglial phagocytosis and reduces synaptic and cellular damage in hippocampal slice cultures [48].

Similarly, TLR4 serves as a central sensor for Aβ-induced neuroinflammatory signaling. In cultured microglia and in the APP/PS1 mouse brain, Aβ triggers the assembly of TLR4 inflammarafts, leading to receptor dimerization, upregulation of TLR4 expression, ROS production, and activation of the NLRP3 inflammasome [18,49]. When microglia were treated with Aβ oligomers, robust increases in lipid raft-localized TLR4 and ROS generation were observed, indicating that Aβ initiates TLR4-driven oxidative stress [18]. Pharmacological inhibition of TLR4 by a selective inhibitor CLI-095 blocked inflammasome activation and prevented IL-1β secretion, thereby protecting HT-22 neurons (mouse hippocampal cell line) from apoptosis induced by microglia-derived conditioned media [49].

In vivo, APP/PS1 mice show elevated brain cytokine levels, which are not observed in APP/PS1 mice carrying TLR4 mutations, further confirming the contribution of TLR4 signaling to Aβ-induced inflammation [50]. Functionally, TLR4 activation is associated with impaired long-term potentiation (LTP) and neuronal injury, effects that are reversed by TLR4 antagonists [51]. Soluble oligomeric Aβ42 at picomolar concentrations gradually sensitizes TLR4 in microglia, elevating TNF-α production over time. These soluble oligomers also impair LTP in hippocampal slices and induce neuronal death in neuron-glia co-cultures—effects rescued by TLR4 inhibition—indicating that TLR4 mediates Aβ-driven neuronal dysfunction through an autocrine/paracrine pathway [51]. Collectively, these results support the notion that TLR4-driven neuroinflammation is a critical mechanism for the pathogenesis of AD.

Mechanistically, full microglial activation in response to fibrillar Aβ requires TLR2, TLR4, and their co-receptor CD14, all localized to lipid rafts. This receptor complex initiates intracellular signaling cascades such as Src-Vav-Rac and p38 MAPK, which are critical for ROS generation and Aβ phagocytosis. Disruption of CD14, TLR2, or TLR4 impairs these responses, underscoring the central role of CD14 in microglial Aβ recognition and response [52]. Consistent with this, Aβ has been shown to activate microglia through various TLR4-containing receptor complexes, including MD-2/TLR4/CD14, TLR2/TLR4/CD14, and TLR4/TLR6/CD36 [42,52,53].

Clinically, a functional polymorphism in the TLR4 gene (Asp299Gly) has been linked to increased longevity and reduced AD risk in several populations. This protective TLR4 variant correlates with improved cognitive performance, increased cortical thickness, and stable cerebrospinal fluid levels of IL-1β over time, suggesting a role for TLR4 signaling modulation in slowing disease progression [54].

### 5.2. CD36

CD36 is a class B scavenger receptor highly expressed in microglia, macrophages and endothelial cells, functioning as an innate immune sensor for endogenous danger signals, including fibrillar Aβ. In AD, CD36 binds directly to Aβ fibrils, triggering a cascade of pathological responses, such as ROS production, vasoconstriction, and disruption of vascular tone, which contribute to neuronal injury and cognitive decline [55,56]. CD36 also plays a central role in microglial activation, where it facilitates the secretion of cytokines and chemokines, thereby promoting microglial migration and recruitment to amyloid plaques [57].

The binding of Aβ to CD36 has been linked to various cellular outcomes depending on the context, with the most common being the production of ROS, pro-inflammatory cytokines, and chemokines that recruit monocytes. These responses have been observed both in cultured microglia and in amyloid-rich regions of the mouse and human brain [58].

Studies with CD36 knockout mice further underscore CD36’s central role in Aβ-induced neuroinflammation. Microglia without CD36 show reduced activation and lower levels of inflammatory cytokines secretion when exposed to Aβ, compared to wild-type microglia. Moreover, direct intracerebral injection of Aβ into CD36-deficient mice induces significantly less microglial accumulation, indicating that CD36 is crucial for the microglial response to amyloid plaques in vivo [57].

At the molecular level, CD36 resides in lipid rafts, where it clusters with co-receptors, including integrins and TLRs, particularly TLR4 and TLR6. Aβ binding induces the formation of a CD36-TLR4-TLR6 complex, which activates downstream MAPK pathways, Src family kinases (e.g., Fyn, Lyn), and pro-inflammatory genes via NF-κB and NLRP3 inflammasome activation [53,55,58]. These signaling events lead to the release of cytokines and ROS, thereby sustaining neuroinflammation and exacerbating AD pathology.

Importantly, the CD36-TLR4-TLR6 receptor complex represents a common signaling axis by which both Aβ and other endogenous ligands, such as oxidized low-density lipoprotein (LDL), stimulate inflammatory pathways [53,59]. While CD36-TLR2/6 complexes are involved in responses to pathogens, the CD36-TLR4/6 module is specifically activated by endogenous danger signals like Aβ, reflecting a ligand-specific receptor assembly that is dependent on lipid rafts [59].

In addition to its role with TLR4/6, CD36 also functions as a co-receptor for TLR2, particularly in association with TLR1. This CD36-TLR2/1 signaling axis mediates post-ischemic neuroinflammation and has been shown to selectively enhance cytokine responses in the central nervous system. CD36-deficient mice display significantly reduced TLR2/1-mediated inflammation following cerebral ischemia, suggesting that CD36 is crucial for TLR2/1 signaling in AD and may contribute to sterile inflammation [60].

Functionally, CD36 serves a dual role in AD progression. In the early stages, it aids in the phagocytosis of Aβ and contributes to its clearance, offering neuroprotection. However, as the disease progresses, this mechanism becomes less efficient. While CD36 expression decreases in chronically activated microglia, the pro-inflammatory output persists, reinforcing a cycle of impaired Aβ clearance and neurodegeneration [58].

Overall, CD36 acts as a critical lipid raft-resident co-receptor that integrates Aβ recognition with inflammatory signaling. CD36 interactions with TLR4/6 and TLR2/1 make it a key amplifier of chronic neuroinflammation in AD, representing a promising therapeutic target for modulating immune responses in neurodegenerative diseases.

### 5.3. RAGE

The receptor for advanced glycation end products (RAGE) belongs to the immunoglobulin superfamily and functions as a pattern recognition receptor capable of binding various ligands, including advanced glycation end products (AGEs), S100B, and Aβ peptides. RAGE is expressed in neurons, glial cells, and brain endothelial cells. In human AD brain tissue, RAGE expression is elevated in neurons, microglia, and endothelial cells, particularly in regions surrounding amyloid plaques. Similarly, in transgenic AD mouse models harboring human APP mutations, RAGE expression increases with age and Aβ accumulation, particularly in neurons and microglia [61,62,63,64].

RAGE is an important binding partner for Aβ and facilitates its translocation across the blood–brain barrier and accumulation within brain parenchyma. Upon binding to Aβ monomers or oligomers, RAGE activates p38 MAPK, ERK1/2, and NF-κB pathways, leading to oxidative stress, mitochondrial dysfunction, upregulation of pro-inflammatory cytokines, and expression of adhesion molecules. These processes contribute to synaptic dysfunction, neuronal damage, and cognitive impairment. Moreover, RAGE-Aβ interaction establishes a pathological positive feedback loop: elevated levels of Aβ at inflammatory sites activate RAGE, inducing oxidative stress and NF-κB activation. NF-κB, in turn, upregulates RAGE expression via response elements in the RAGE gene promoter, thereby sustaining chronic inflammation and enhancing Aβ-related neurotoxicity [61,62,65].

Recent studies have revealed that RAGE forms homodimers within lipid rafts—a process facilitated by S100B and AGEs—which is essential for high-affinity ligand recognition and downstream signal transduction [63].

Functional studies in AD mouse models support the causative role of RAGE in AD pathogenesis. Co-expression of mutant APP and RAGE (mAPP/RAGE) results in increased Aβ levels and amyloid plaque burden in the cortex and hippocampus compared to mAPP expression alone. Conversely, expression of a dominant-negative RAGE mutant (DN-RAGE), which lacks signal transduction capacity, mitigates Aβ accumulation, glial activation, and cognitive deficits in these models [61].

### 5.4. TREM2

TREM2 is a transmembrane receptor expressed in the brain almost exclusively in microglia, where it plays a crucial role in neuroinflammation and maintaining neuronal homeostasis [66]. In AD, TREM2 expression is notably upregulated, particularly in microglia surrounding Aβ plaques [67]. Elevated soluble TREM2 (sTREM2) levels in cerebrospinal fluid of AD patients have been suggested as a biomarker for microglial activation and neuroinflammation [68].

TREM2 is a receptor located within lipid rafts, where it is associated with DAP12, and plays a role in the recognition of Aβ [69]. TREM2 specifically binds oligomeric Aβ1-42 with high affinity, triggering downstream signaling pathways that influence microglial behavior. This interaction promotes microglial migration toward and clustering around Aβ plaques. Mutations in the TREM2 gene, such as the R47H variant, have been linked to an increased risk of late-onset AD and reduced binding affinity for Aβ, which may impair the microglial response to amyloid pathology. These mutations suggest a crucial role for TREM2 in mediating the interaction between microglia and Aβ, facilitating immune responses that help control plaque accumulation and neuroinflammation [70].

TREM2 activation triggers the immunoreceptor tyrosine-based motif (ITAM) pathway, leading to microglial activation. This response is consistent with the upregulation of TREM2, along with other microglial activation markers such as APOE and TYROBP during neuroinflammation [41]. In AD mouse models, overexpression of TREM2 has been shown to enhance the expression of genes associated with phagocytosis, suggesting a protective role in clearing amyloid plaques. However, in some cases, excessive TREM2 activation has been linked to neuroinflammation and disruption of microglial homeostasis, particularly through APOE-dependent signaling. This dual role underscores the complexity of TREM2 involvement in neurodegenerative processes, where its effects may vary depending on the disease stage and microglial state [41]. For instance, in AD models, TREM2 deficiency impairs microglial clustering around Aβ plaques, exacerbating disease pathology by reducing Aβ clearance and increasing neuroinflammation [71,72].

Given its critical role in AD, TREM2 has emerged as a potential therapeutic target. Modulating TREM2 signaling, e.g., via regulation of lipid rafts, could help enhance microglial responses to Aβ or reduce neuroinflammation, but strategies must be tailored to disease stage and microglial state [41].

### 5.5. Cholesterol

Cholesterol is a fundamental component of cellular membranes, enriched in lipid rafts, where it modulates membrane fluidity, protein localization, and signal transduction. In AD, cholesterol plays a pivotal role in the synthesis, aggregation, and internalization of Aβ. The cholesterol-rich microdomains provide an optimal environment for the amyloidogenic processing of APP, as β- and γ-secretases localize to lipid rafts and exhibit enhanced activity in cholesterol-rich conditions [24,73,74]. Conversely, high cholesterol inhibits α-secretase [75]. Experimental depletion of cholesterol has been shown to disrupt lipid rafts, increase membrane fluidity, promote α-secretase ADAM10 activity [74], and reduce Aβ production [73].

Cholesterol also increases lipid packing and surface hydrophobicity, features that facilitate Aβ membrane binding, seeding, and toxicity. Molecular dynamics simulations and in vitro studies demonstrate that Aβ preferentially associates with cholesterol and GM1 within these domains, forming early aggregation complexes [24,73]. Lipid rafts thus serve not only as catalytic sites for Aβ generation but also as key regions for its extracellular binding and internalization. Oligomeric Aβ interacts with gangliosides and sphingomyelin in these domains, triggering fibrillization, membrane disruption, oxidative stress, and a positive feedback loop that enhances further APP cleavage. The internalization of Aβ oligomers through GM1-mediated, cholesterol-dependent endocytosis further underscores the centrality of lipid rafts in AD pathology [9,73]. Notably, disrupting rafts or reducing cholesterol content attenuates Aβ-induced toxicity [73], while moderate cholesterol levels may offer protection against membrane damage by Aβ-derived diffusible ligands (ADDLs) [74].

Midlife hypercholesterolemia is consistently associated with greater Aβ burden and increased risk of AD [9,73]. High-cholesterol diets accelerate amyloid pathology, while statin treatment or cholesterol-lowering interventions often mitigate AD features, although clinical outcomes remain mixed [27,73]. Experimental studies further show that a 30% increase in cellular cholesterol enhances Aβ toxicity, whereas a similar reduction diminishes it [24]. In the aging or AD brain, changes in lipid composition—marked by reduced levels of unsaturated fatty acids and elevated cholesterol—result in increased lipid raft number and rigidity. These altered lipid raft properties facilitate the accumulation of APP and strengthen its interaction with β-secretases [27].

Intriguingly, Aβ itself perturbs cholesterol homeostasis. Toxic Aβ oligomers, but not monomers, raise synaptic cholesterol levels by enhancing cholesterol ester hydrolysis. This shift results in reduced cholesterol ester content and elevated free cholesterol, likely through activation of cholesterol ester hydrolase (CEH) [76]. Blocking CEH reverses this effect, implicating a specific mechanism by which Aβ alters membrane composition and promotes synaptic dysfunction—potentially via clustering of membrane receptors such as the cellular prion protein (PrPc) [76].

Cholesterol also directly interacts with β-CTF, the β-secretase product of APP, to further promote amyloidogenic processing and Aβ generation. On the other hand, cholesterol depletion with methyl-β-cyclodextrin (MβCD) reduces APP processing and AICD production, which in turn decreases the transcription of several neuronal genes, especially the Aβ-degrading enzyme, neprilysin [74]. This illustrates the delicate balance between cholesterol levels and Aβ homeostasis.

Furthermore, Aβ peptides influence lipid metabolism by modulating cholesterol esterification and phospholipid turnover, while also impairing vesicle trafficking and contributing to cholesterol accumulation in the Golgi and plasma membrane [74,76]. Aβ40 specifically inhibits HMG-CoA reductase, the rate-limiting enzyme in cholesterol biosynthesis, and AICD regulates cholesterol uptake via lipoprotein receptors like LRP1 [77]. These findings support the notion that Aβ functions as part of lipoprotein complexes, reshaping local and systemic lipid dynamics.

Altogether, the interplay between cholesterol and Aβ spans multiple facets of AD pathology—from APP processing and membrane integrity to vesicle trafficking and lipid metabolism. Therapeutic approaches targeting cholesterol content or distribution, particularly within lipid rafts, may hold promise [73,74]. This includes potential interventions to disrupt Aβ oligomerization, reduce lipid raft-dependent internalization, or rebalance cholesterol metabolism. However, given cholesterol’s essential roles in neuronal function, such interventions must aim for selective and precise modulation rather than broad suppression.

### 5.6. Gangliosides

Gangliosides are sialic acid-containing glycosphingolipids anchored in the outer leaflet of the plasma membrane, where they participate in membrane structure and cellular signaling. Neuronal cells are particularly enriched in gangliosides, which contribute to the organization of lipid rafts and interactions with membrane proteins [74]. Disruption of ganglioside metabolism leads to profound neurological consequences, as demonstrated in knockout mice with phenotypes mimicking neurodegenerative disorders including Alzheimer’s and Parkinson’s disease [78,79]. In aging and especially in AD, overall ganglioside content declines in several brain regions relevant to disease pathology [80]. Interestingly, despite this overall reduction, specific gangliosides such as GM1 and GM2 are found to accumulate in lipid rafts of AD brains [81].

Aβ oligomers exhibit high affinity for GM1 ganglioside, especially in lipid rafts, facilitating their aggregation into toxic β-sheet-rich fibrils [74,82]. The GM1-Aβ complex has been found in the AD brain and serves as a seed for further Aβ aggregation [74,82,83]. Cholesterol enhances GM1 clustering within lipid rafts, promoting Aβ seeding [74]. These GM1 clusters provide a hydrophobic platform that enables Aβ conformational changes and oligomerization, contributing to LTP impairment and neurotoxicity [84]. Disruption of ganglioside synthesis leads to decreased Aβ-induced neurotoxicity and improved memory in AD model mice [82].

Recent therapeutic approaches target the GM1-Aβ interaction. The synthetic peptide AmyP53, derived from Aβ and α-synuclein ganglioside-binding domains, competitively binds GM1 and prevents Aβ from initiating neurotoxic cascades [85]. AmyP53 exhibits blood–brain barrier permeability and holds promise as a therapeutic agent for AD and Parkinson’s disease [86].

### 5.7. Sphingomyelin

Sphingomyelin (SM), a major component of lipid rafts, is particularly enriched in the brain and peripheral nervous tissue, where it plays essential roles in development, differentiation, and immune responses. SM is crucial for the activity of several membrane receptors, such as NMDA, α7 nicotinic, 5-HT1A, and TrkB receptors [74]. In AD, SM metabolism is significantly altered. Lipidomic studies show decreased SM and increased ceramide levels in AD brains, likely due to enhanced sphingomyelinase (SMase) activity, which disrupts lipid raft formation and impairs GLUT4 function, contributing to energy metabolism deficits [74,87].

Additionally, Aβ accumulation in neurons activates SMase, leading to SM depletion and abnormal APP processing [74]. Toxic Aβ42 peptides can further enhance SMase activity, promoting early SM loss in AD and disrupting protein–lipid interactions and downstream signaling [88]. Notably, APP, Aβ, and other pathogenic proteins like gp120 and PrP share SM-binding motifs, emphasizing the central role of SM in mediating neurodegenerative processes [89]. Acid SMase activity correlates with Aβ42 levels in cognitively normal individuals, a relationship lost in AD, suggesting impaired SM metabolism may hinder Aβ clearance [90]. Together, these findings highlight a complex, compartment-specific dysregulation of SM and SMase activity in AD pathogenesis.

## 6. Inflammarafts as a Therapeutic Target

Traditional therapeutic strategies have primarily focused on targeting Aβ, the hallmark pathological peptide in AD. Anti-amyloid disease-modifying therapies fall into three main categories: inhibition of Aβ42 production, prevention of Aβ oligomerization, and enhancement of Aβ clearance via immunotherapy [91]. Despite extensive efforts, the majority of clinical trials targeting Aβ have been unsuccessful [9]. Monoclonal antibodies such as aducanumab and lecanemab have demonstrated efficacy in reducing plaque burden and modestly slowing cognitive decline. However, these benefits are often overshadowed by adverse events such as amyloid-related imaging abnormalities, cerebral edema and microhemorrhage, which limit their widespread clinical utility and require careful patient selection and monitoring [92].

The limitations of Aβ directed therapies necessitate identification of novel therapeutic targets. A selective lipid raft therapy, specifically targeting the lipid rafts involved in Aβ production and the microglial inflammarafts that organize Aβ-induced neuroinflammatory processes, may afford new opportunities in treatment of AD. Selectivity of such a therapy toward inflammarafts would ensure that the integrity of physiological lipid rafts is preserved and preclude adverse side effects.

### 6.1. Modulators of Sphingolipid Metabolism

Modulating cellular membrane lipid composition and lipid raft biophysical organization has emerged as a potential therapeutic strategy. Disruption of sphingolipid synthesis, via serine palmitoyl transferase inhibition or subunit mutations, alters Aβ metabolism by enhancing non-amyloidogenic cleavage [93]. Furthermore, therapeutic interventions targeting gangliosides such as GM1 have shown promise. Intraventricular administration of GM1 in AD patients was reported to halt cognitive decline and improve both motor and neuropsychological functions [94]. Peripheral delivery of GM1 has also been proposed to modulate Aβ dynamics between the periphery and the brain, thereby reducing cerebral Aβ burden in transgenic mouse models [95]. Nevertheless, the immunogenic nature of gangliosides may limit clinical application due to the potential for adverse immune responses [74].

### 6.2. Modulators of Cholesterol Metabolism

Similarly, cholesterol-lowering agents such as statins have been evaluated for their capacity to disrupt lipid rafts by inhibiting HMG-CoA reductase and reduce cholesterol synthesis. Although in vitro studies suggest that moderate cholesterol depletion may suppress Aβ production, clinical outcomes have been inconsistent, and cognitive improvement has not been reliably demonstrated [74]. These discrepancies may stem from differences in treatment timing and the extent of cholesterol reduction; modest depletion (<25%) may preserve membrane integrity and be neuroprotective, whereas more extensive depletion (>35%) can impair cellular functions [10]. Agents like MβCD are highly effective in extracting membrane cholesterol and disrupting raft structures in vitro, but their systemic toxicity and disruption of cellular homeostasis preclude their wide therapeutic use in humans.

Cholesterol distribution within cells also influences AD pathology. Alterations in subcellular cholesterol distribution have been shown to modulate APP processing. For example, impaired cholesterol transport from late endosomes to the endoplasmic reticulum by U18666A (amphipathic amino-steroid) reduces Aβ secretion [10,96]. Similarly, enhanced cholesterol efflux, through activation of liver X receptors (LXRs) that upregulate the downstream target ATP-binding cassette transporter A1 (ABCA1), also reduces Aβ generation by inhibiting BACE1 and γ-secretase cleavage of APP [97].

Overall, the lack of selectivity in reducing cholesterol content by statins or MβCD and ensuing disruption of physiological lipid rafts and associated toxicity impede the application of these strategies in therapy for AD.

### 6.3. AIBP

To address the shortcomings of non-selective cholesterol modulators, recent studies have identified apolipoprotein A-I binding protein (AIBP) as a selective regulator of lipid raft function. AIBP facilitates cholesterol efflux specifically in activated cells, targeting inflammarafts due to its binding to TLR4, the receptor upregulated in inflammatory cells. By promoting cholesterol efflux, AIBP destabilizes inflammarafts, reduces TLR4 clustering, and suppresses cytokine production [39,40]. In contrast to statins or MβCD, AIBP acts selectively in inflamed or cholesterol-overloaded cells, sparing non-inflammatory cells and preserving physiological lipid rafts.

In endothelial cells, AIBP triggers re-localization of γ-secretase from lipid rafts to non-lipid raft domains where it cleaves Notch (receptor involved in cell proliferation and differentiation pathways) [98]. If AIBP has a similar effect on γ-secretase transition from lipid rafts where it co-localizes with APP to non-lipid raft domains in neurons, this will inhibit Aβ production.

Experimental evidence demonstrates that intrathecal administration of AIBP effectively suppresses neuroinflammation in animal models without impairing sensory or motor function [39]. This suggests that AIBP selectively targets inflammarafts in activated immune or glial cells while saving normal physiological signaling in non-inflammatory cells. In a cisplatin-induced neuropathic pain model, a single intrathecal injection of AIBP not only reversed established mechanical allodynia but also maintained its analgesic effect for as long as two months [39]. Such long-lasting efficacy from a single dose underscores AIBP’s therapeutic potential in chronic neuroinflammatory conditions, offering a novel approach that combines specificity, potency, and safety.

In the context of neurodegenerative diseases, AIBP also demonstrates protective effects by reducing Aβ-induced microglial activation, lipid raft formation, and ROS generation, thereby attenuating both neuroinflammation and oxidative stress [18]. The AIBP deficiency exacerbates AD-related neuropathology; in APP/PS1 mouse models, loss of AIBP results in increased Aβ plaque burden, microglial activation and increased neuronal cell death [18]. Given the complex interplay between cholesterol metabolism, microglial function, and Aβ pathology, AIBP’s ability to selectively modulate inflammarafts presents a promising therapeutic avenue. Continued investigation into its efficacy and safety in chronic neurodegenerative models is warranted, particularly through upregulation of AIBP function studies in AD mouse models.

## 7. Conclusions

Aβ remains a central challenge in Alzheimer’s disease research and continues to be a highly active area of investigation. Beyond traditional Aβ-targeting therapies, there is growing interest in exploring alternative pathways, particularly those that target inflammarafts—enlarged lipid raft structures involved in neuroinflammation. This review has highlighted the role of protein receptors and lipid components within lipid rafts, emphasizing their contribution to AD pathology. Large-scale genome-wide association studies of late-onset AD have uncovered several genetic risk alleles associated with immune and inflammatory responses, many of which are highly expressed in microglia, highlighting the pivotal role of microglial inflammation in AD pathogenesis. However, designing targeted therapies for each individual genetic risk allele is costly and time-consuming. This reinforces the relevance of targeting inflammarafts to modulate overall microglial responses and mitigate neuroinflammation.

Among potential therapeutic options, AIBP, with its prolonged therapeutic effect and selective action, emerges as a promising candidate for targeting inflammarafts. However, while current evidence is promising, further research and development are crucial to fully elucidate the therapeutic potential of AIBP and its role in targeting inflammarafts. In addition, chronic inflammation is a common feature not only in neurodegenerative diseases but also in various other pathological conditions such as glaucoma, neuropathic pain, atherosclerosis, and asthma [99,100,101,102]. Given its broad applicability, targeting inflammarafts could represent a novel therapeutic strategy with wide-ranging implications for improving patient health across multiple diseases.
